# TNFα Induces DNA and Histone Hypomethylation and Pulmonary Artery Smooth Muscle Cell Proliferation Partly via Excessive Superoxide Formation

**DOI:** 10.3390/antiox13060677

**Published:** 2024-05-31

**Authors:** Patrick Crosswhite, Zhongjie Sun

**Affiliations:** 1 Department of Physiology, College of Medicine, University of Oklahoma Health Sciences Center, Oklahoma City, OK 73104, USA; 2 Department of Human Physiology, Gonzaga University, Spokane, WA 99205, USA; 3 Department of Physiology, College of Medicine, University of Tennessee Health Sciences Center, Memphis, TN 38163, USA

**Keywords:** TNF-α, DNA methylation, histone methylation, GADD45-α, proliferation, pulmonary arterial smooth muscle cells

## Abstract

**Objective:** The level of tumor necrosis factor-α (TNF-α) is upregulated during the development of pulmonary vascular remodeling and pulmonary hypertension. A hallmark of pulmonary arterial (PA) remodeling is the excessive proliferation of PA smooth muscle cells (PASMCs). The purpose of this study is to investigate whether TNF-α induces PASMC proliferation and explore the potential mechanisms. **Methods:** PASMCs were isolated from 8-week-old male Sprague-Dawley rats and treated with 0, 20, or 200 ng/mL TNF-α for 24 or 48 h. After treatment, cell number, superoxide production, histone acetylation, DNA methylation, and histone methylation were assessed. **Results:** TNF-α treatment increased NADPH oxidase activity, superoxide production, and cell numbers compared to untreated controls. TNF-α-induced PASMC proliferation was rescued by a superoxide dismutase mimetic tempol. TNF-α treatment did not affect histone acetylation at either dose but did significantly decrease DNA methylation. DNA methyltransferase 1 activity was unchanged by TNF-α treatment. Further investigation using QRT-RT-PCR revealed that GADD45-α, a potential mediator of DNA demethylation, was increased after TNF-α treatment. RNAi inhibition of GADD45-α alone increased DNA methylation. TNF-α impaired the epigenetic mechanism leading to DNA hypomethylation, which can be abolished by a superoxide scavenger tempol. TNF-α treatment also decreased H3-K4 methylation. TNF-α-induced PASMC proliferation may involve the H3-K4 demethylase enzyme, lysine-specific demethylase 1 (LSD1). **Conclusions:** TNF-α-induced PASMC proliferation may be partly associated with excessive superoxide formation and histone and DNA methylation.

## 1. Introduction

A majority of pulmonary arteries have three essential layers: (1) the outer adventitia composed primarily of fibroblasts, (2) a media of smooth muscle cells and elastic laminae, and (3) the inner intima composed of a single layer of endothelial cells. Pulmonary hypertension (PH) can be described as a remodeling disease characterized by medial thickening and concentric intimal fibrosis [1]. Excessive PASMC proliferation and distal extension of smooth muscle into peripheral, normally non-muscular, pulsmonary arteries are typical characteristics of PH [1,2]. In fact, the understanding of PH has transitioned from a model of vasoconstriction and impaired vasodilation to one now defined by excess growth and proliferation of abnormal cells. In our experiments, we have repeatedly shown that cold exposure causes PASMC proliferation and vascular remodeling [3,4,5], but mechanisms of the cold-induced PASMC proliferation, however, have not been investigated. Over-proliferation of PASMCs and the consequent pulmonary arterial remodeling are also found in other models of PH and patients with PH [1]. Thus, excessive PASMC proliferation is a hallmark of PH [1]. However, the underlying mechanism of over-proliferation of PASMCs in PH is not fully understood.

While the vast majority (70%) of epigenetic publications are related to a variety of cancers, there is a rising body of evidence that epigenetic mechanisms are involved in PH [6]. The term epigenetics is used to describe all heritable changes in phenotype or gene expression states that are not due to changes in the DNA sequence [7,8]. Epigenetic modifications provide a mechanism that allows the stable propagation of gene activity states from one generation to the next [9]. Epigenetics might also be able to address unexplained observations in PH. For example, although most cases of familial PH involve bone morphogenetic protein receptor (BMPR2) mutations, it is still unknown why only 20% of BMPR2 carriers ever develop the disease. The main mechanisms of epigenetic modification include RNA interference, histone modifications, and DNA methylation, all of which are able to regulate a variety of cellular mechanisms, including proliferation [1].

Histone modifications include a variety of mechanisms including histone acetylation, methylation, phosphorylation, ubiquitination, and others. Histones are the core particle proteins of the nucleosome that accommodate 147 base pairs of DNA and are an integral component of the mechanisms responsible for the regulation of gene transcription [10]. Normally, the N-terminal tails of histones are modified and result in structural changes in the nucleosome. These changes can include the relaxation of the nucleosome to allow transcriptional element binding and increase transcription activity, or can result in a more compact nucleosome that prevents transcriptional element binding, therefore preventing transcription activity [11]. Acetylation is one of the most frequent histone modifications and occurs when an acetyl group is added to a lysine residue located on a histone tail. The main sites of histone acetylation include lysine (K)9, K14, K18, and K23 on histone H3 while K5, K8, K12, and K16 are common on histone H4 [7,8]. Increased histone acetylation is highly correlated with an increase in gene transcription [12,13,14]. Like acetylation, histone methylation also occurs on the lysine residues. The covalent attachment of a methyl group on a lysine, however, may either activate or repress gene transcription, depending on the site of methylation [10]. For example, methylation of K9 and K27 on histone H3 is associated with transcriptional silencing, while methylation of K4, K36, and K79 is associated with increased gene activity [10,15]. It is not known, however, whether histone modification is involved in PASMC proliferation.

DNA methylation, the addition of a methyl group to the C5 position of cytosine, is an essential process for mammalian development involved in many cellular processes. The presence of CpG islands, or concentrated areas where the cytosine and guanine residues are next to each other in sequence, is the most well-studied DNA methylation phenomenon. CpG islands are typically located in promoter or enhancer regions of genes and the methylation status of these areas can alter gene transcription. In general, hypermethylation of CpG islands is associated with gene silencing, whereas hypomethylation is associated with gene overexpression. In the context of SMC proliferation, either the silencing of tumor suppressor genes or the overexpression of tumor-promoting genes could contribute to uncontrolled cell growth and proliferation [16,17,18]. A family of enzymes called DNA methyltransferases, or DNMTs, regulate the methylation status of a gene. The DNMT1 enzyme generally regulates maintenance methylation, while DNMT3a and DNMT3b appear to regulate de novo methylation [6].

It is well established that environmental factors influence one’s genome and gene expression profile [19,20,21,22,23,24,25]. The cold-induced pulmonary hypertension (CIPH) model is an environmentally induced form of PH [3,4,5] that has clinical importance for humans who live in cold regions or who work outside during the winter months. We have established that cold exposure increases TNF-α expression and contributes to CIPH and pulmonary arterial remodeling [5]. Upregulation of TNF-α in pulmonary arteries and lungs is also found in other animal models of PH [1], as well as in PH patients [26,27]. It is not known, however, whether TNFα directly stimulates PASMC proliferation and what molecular mechanism mediates this process. To address this gap in knowledge, we hypothesize that PASMCs treated with exogenous TNF-α alter epigenetic mechanisms that promote cell proliferation.

TNF-α is a powerful inflammatory cytokine that impairs vascular cell function [28]. NADPH oxidase is the major source of vascular superoxide [29,30,31,32]. In this study, we will determine whether TNF-α may affect NADPH oxidase activity and superoxide production in PASMCs. We will further investigate the potential downstream epigenetic mechanisms involving TNF-α-induced PASMC proliferation.

## 2. Methods and Materials

**Isolation of PASMCs.** PASMCs were isolated from rat pulmonary arteries as described in our recent study [5]. Briefly, we isolated PASMCs from Sprague-Dawley rats (150–180 g) that were maintained at room temperature (23.5 ± 0.5 °C). The procedure was approved by the OUHSC Institutional Animal Care and Use Committee (IACUC). PASMCs from passages 1–5 were used for the following cell culture procedures.

**TNF-α and PASMC Proliferation.** Cell proliferation was carried out as described in our previous studies [30,33,34,35,36,37,38,39,40,41]. PASMCs were seeded in 6-well plates (5 × 10^4^/well) in culture media (DMEM #12430, 10% fetal bovine serum (FBS), and 1% penicillin/streptomycin, Life Technologies, Carlsbad, CA, USA) and allowed to attach overnight at 37 °C. In low-serum conditions (0.1% FBS), recombinant rat TNF-α (R&D Systems, Minneapolis, MN, USA) was added to wells at a concentration of 0, 20, or 200 ng/mL for 24 and 48 h (media and TNF-α were refreshed after 24 h) for proliferation studies. After TNF-α incubation, the cells were trypsinized (0.25%, Life Technologies), collected, and resuspended in 1 mL media and 10 μL samples (min. 5 samples) were used to assess the total number of cells using an automated cell counter (TC-10, Bio-Rad Laboratories, Hercules, CA, USA). Cells were also counted individually using phase contrast images taken of live cells in culture. Briefly, a minimum of five images were taken of two different wells for each treatment (0, 20, 200 ng TNF-α) for each time point (24 and 48 h). Cells were counted according to nuclei and the average number of cells per photograph was determined.

**NADPH Oxidase Activity.** NADPH oxidase activity in PASMCs was measured using the lucigenin chemiluminescence method as we described previously [29,42,43].

**Superoxide Production in PASMCs.** The detailed procedure for measuring superoxide production in PASMCs was adapted from our previous work [30,34,44,45]. Briefly, PASMCs were seeded in 6-well plates (5 × 10^4^/well) in culture media and treated with TNF-α for 24 and 48 h. After 24 h, the cells were rinsed 2× with ice-cold PBS and then DHE (10 μM, Sigma-Aldrich, St. Louis, MO, USA) was allowed to incubate for 30 min at 37 °C in the dark. After 30 min, the excess DHE was rinsed away using PBS and the nuclear stain DAPI (Santa Cruz Biotechnology Inc., Santa Cruz, CA, USA) was allowed to incubate for 5 min at room temperature in the dark. Superoxide production was immediately accessed using a Leica TCS NT Confocal fluorescence microscope. This procedure was then repeated for the 48-h TNF-α treatment.

**Total Histone H3 Acetylation Determination.** Using an ELISA-based kit specific for acetylated H3 histone proteins (#P-4030, Epigentek Group Inc., Farmingdale, NY, USA), we determined the total amount of H3 acetylation in PASMCs after 24 and 48 h treatment with TNF-α. Histone protein was extracted prior to the measurement as directed by the manufacturer.

**Global DNA Methylation Determination.** DNA was isolated from PASMCs treated with TNF-α (for 24 and 48 h) using a DNA isolation kit (#P-1018, Epigentek, Farmingdale, NY, USA). After isolating DNA, global DNA methylation was determined by measuring levels of 5-methylcytosine (5-mC) in an ELISA-based microplate format (#P-1034, Epigentek, Farmingdale, NY, USA).

**Real-time Polymerase Chain Reaction (RT-PCR).** The real-time RT-PCR was performed as described previously [33,45,46,47,48,49,50]. Briefly, total RNA was isolated from PASMCs treated with or without TNF-α (0, 20, or 200 ng/mL) for 24 or 48 h. Several nucleotide or base excision repair genes attributed to the demethylation of DNA were evaluated using a Bio-Rad CFX96-C1000 thermal cycler (Bio-Rad Laboratories, Hercules, CA, USA). GAPDH was used to compare the relative mRNA expression of all genes. Supplemental Table S1 lists all primers used.

**Tempol-treated PASMCs.** PASMCs were seeded in 6-well plates (5 × 10^4^/well) in culture media (DMEM #12430 Life Technologies, 10% FBS, and 1% penicillin/streptomycin) and allowed to attach overnight at 37 °C. In low-serum conditions, PASMCs were pre-treated with or without the anti-oxidant tempol (4-hydroxy-Tempo, Sigma-Aldrich Co., St. Louis, MO, USA) overnight. The media was then replaced with low-serum and treated with TNF-α (0, 20, or 200 ng/mL) for 24 and 48 h (media, tempol, and TNF-α were refreshed after 24 h). PASMC proliferation, superoxide production, and DNA methylation were then determined as described above.

**Histone (H3-K4) Methylation Measurement in PASMCs.** PASMCs were seeded in 6-well plates (5 × 10^4^ cells/well) in culture media and allowed to attach overnight at 37 °C. In low-serum conditions, PASMCs were treated with TNF-α for 48 h. Histone proteins were then extracted from the PASMCs and the total histone H3 methylation status of lysine residue 4 (H3-K4) was determined using an ELISA-based kit specific for methylated H3-K4 residues (#P-3017 Epigentek, Farmingdale, NY, USA).

**Pargyline-treated PASMCs.** PASMCs were seeded in 6-well plates (5 × 10^4^/well) in culture media (DMEM #12430, 10% FBS, and 1% penicillin/streptomycin, Life Technologies, Carlsbad, CA, USA) and allowed to attach overnight at 37 °C. In low-serum conditions, PASMCs were co-treated with or without the histone demethylase inhibitor pargyline (Sigma-Aldrich Co., St. Louis, MO, USA) and with or without TNF-α (20 or 200 ng/mL) for 48 h (media, pargyline, and TNF-α was refreshed every 24 h). PASMC proliferation, superoxide production, and histone demethylase activity were then determined.

**Determination of Histone Demethylase Activity in PASMCs.** Histone proteins were extracted from the PASMCs co-treated with pargyline and TNF-α. The activity of the histone demethylase inhibitor LSD1, or lysine-specific demethylase 1, was then determined using an ELISA-based kit (#P-3078 Epigentek, Farmingdale, NY, USA) and a microplate reader.

**Statistical Analysis.** Data were analyzed using a two-way ANOVA (doses and times) followed by the Newman–Kurls procedure. Data = means ± SEM. A probability value with *p* < 0.05 was considered significant.

## 3. Results

### 3.1. TNF-α Treatment Increases Superoxide Production in PASMCs

**Figure 1:**
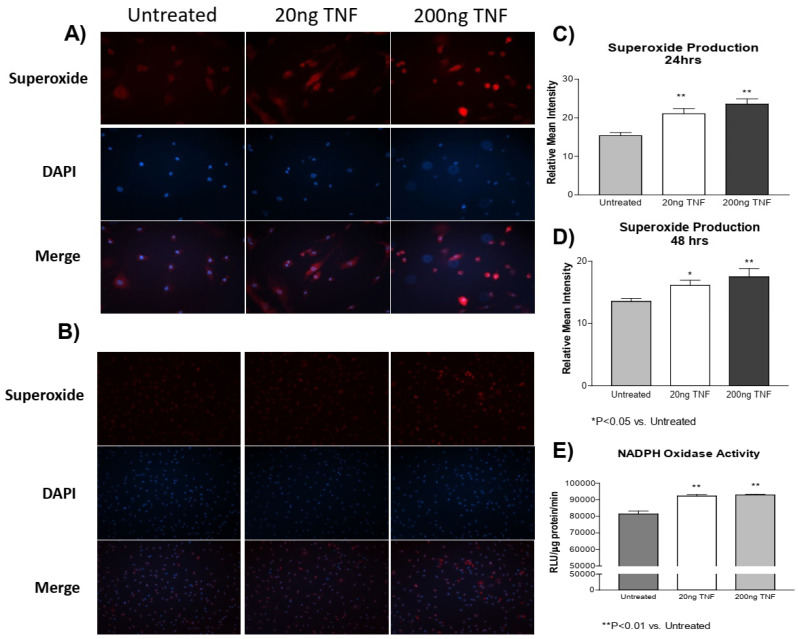
**TNF-α treatment increases superoxide production in PASMCs.** PASMCs were treated with recombinant TNF-α (rTNF-α) for 24 or 48 h. The cells were then incubated with dihydroethidium (DHE), rinsed, and the nuclear stain DAPI was applied. Cell lysates were also collected and NADPH oxidase activity was measured using a lucigenin assay. (**A**) Photos showing representative superoxide production (**red**), nuclear staining (**blue**), and the merged images at 24 h of treatment (200×). (**B**) Photos showing representative superoxide production (**red**), nuclear staining (**blue**), and the merged images at 48 h of treatment (100×). (**C**) Quantification of superoxide production in PASMCs at 24 h of treatment. (**D**) Quantification of superoxide production in PASMCs at 48 h of treatment. (**E**) NADPH oxidase activity at 24 h of treatment of treatment. *n* = 3 independent replicates. * *p* < 0.05 and ** *p* < 0.01 vs. untreated.

In a previous study [4], we showed that cold exposure increased TNF-α expression and superoxide production in both the PA and isolated PASMCs. However, whether TNF-α induces superoxide production in PASMCs is not clear. Here, we found that recombinant rat TNF-α protein treatment increased superoxide production in PASMCs at both 24 and 48 h compared to the untreated control cells (Figure 1A–D). Furthermore, we also found that NADPH oxidase activity was increased due to TNF-α treatment (Figure 1E). These observations suggest that the TNF-α-induced increase in superoxide generation is mediated by activation of NADPH oxidase.

### 3.2. TNF-α Treatment Increases PASMC Proliferation

**Figure 2:**
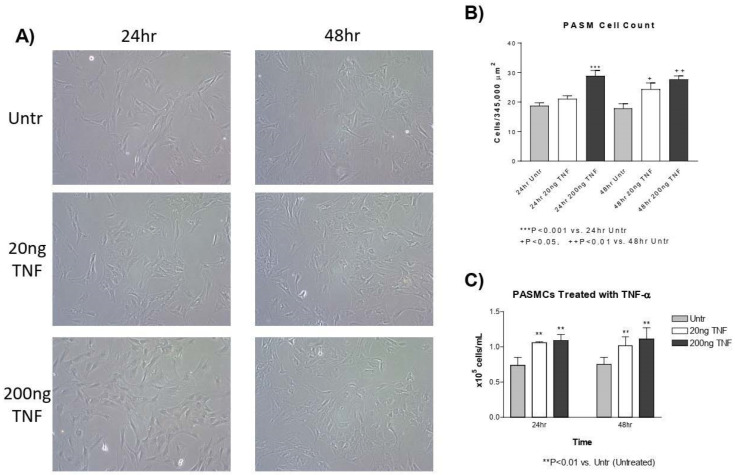
**TNF-α treatment increases PASMC proliferation**. (**A**) PASMCs were treated with rTNF-α for 24 and 48 h and cell proliferation was assessed using two different methods. (**B**) The average number of cells was counted in each photographic field (5 photos per well, 3 wells/treatment). (**C**) The number of cells counted using an automated Bio-Rad cell counter. *n* = 3 independent replicates. ** *p* < 0.01, *** *p* < 0.001 vs. 24 h untreated; ^+^ *p* < 0.05 and ^++^ *p* < 0.01 vs. 48 h untreated. Photos are shown at 200×.

Next, we assessed whether treatment with TNF-α increases the proliferation of isolated PASMCs using two different approaches. First, images of TNF-α treated (20 or 200 ng/mL) or untreated PASMCs were taken 24 and 48 h after plating using phase contrast imaging (Figure 2A). The numbers of cells were counted for each photo and the average number of cells per image was established. At 24 h, there was a significant increase in the number of PASMCs treated with TNF-α compared to the untreated control (Figure 2A,B). At 48 h, there was also a significant increase in the number of PASMCs treated with both 20 ng/mL and 200 ng/mL TNF-α (Figure 2A,B). To confirm this, we used an automated cell counter to determine the cell number. At 24 and 72 h after treatment with TNF-α, there was a significant increase in the number of PASMCs (Figure 2C). Collectively, TNF-α causes PASMC proliferation.

### 3.3. Tempol Prevents TNF-α-Induced Superoxide Increase and PASMC Proliferation

**Figure 3:**
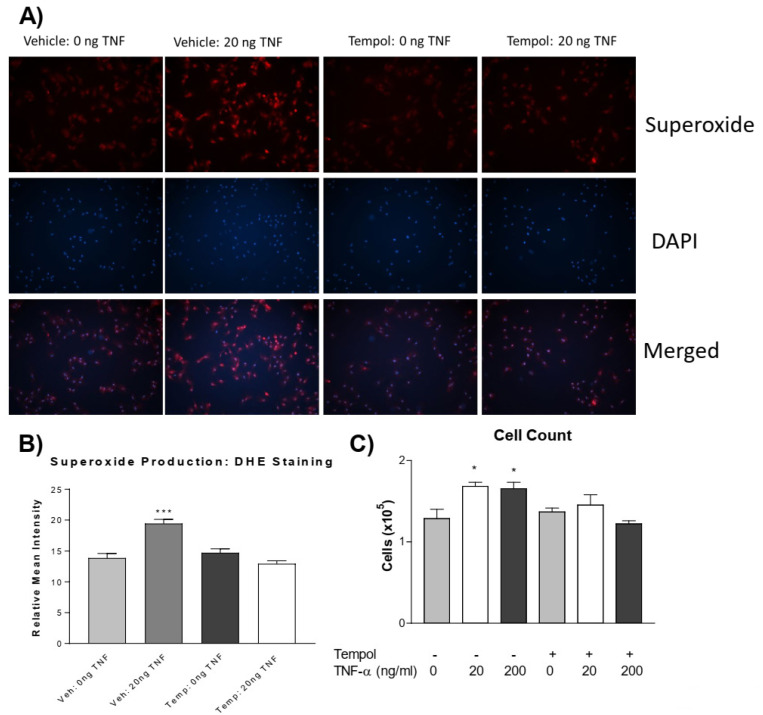
**Tempol prevents TNF-α induced superoxide increase and PASMC proliferation.** PASMCs were pre-treated with or without tempol (1 mM) for 24 h followed by treatment with rTNF-α for 24 h. (**A**) Photos from the DHE staining, as described previously, showing the superoxide production (red), nuclear staining (blue), and merged images. (**B**) Superoxide level. (**C**) Cell proliferation assessed using an automated cell counter. Tempol was dissolved in DMEM (vehicle) immediately before adding to cell culture dishes. *n* = 3 independent replicates. * *p* < 0.05, *** *p* < 0.001 vs. Vehicle 0 ng. Photos are shown at 100×.

We further investigated whether increased superoxide mediates PASMC proliferation. Using the superoxide dismutase (SOD) mimetic, 4-hydroxy TEMPO (tempol), we tested whether the quenching of superoxide could prevent TNF-α-induced PASMC proliferation. Pre-treatment with tempol abolished the TNF-α-induced increase in superoxide levels (Figure 3A,B). Interestingly, tempol also prevented TNF-α-induced PASMC proliferation (Figure 3C). Therefore, TNF-α-induced PASMC proliferation is mediated by superoxide.

### 3.4. TNF-α Treatment Does Not Alter Histone Acetylation in PASMCs

We hypothesized that TNF-α treatment in PASMCs alters epigenetic mechanisms that result in proliferation. To investigate this, we first determined the global acetylation status of histone H3. Unexpectedly, TNF-α treatment did not significantly increase or decrease H3 acetylation in PASMCs (Figure S1A–C), which suggests that H3 acetylation is not involved in TNF-α induced PASMC proliferation.

### 3.5. TNF-α Treatment Decreases DNA Methylation in PASMCs

**Figure 4:**
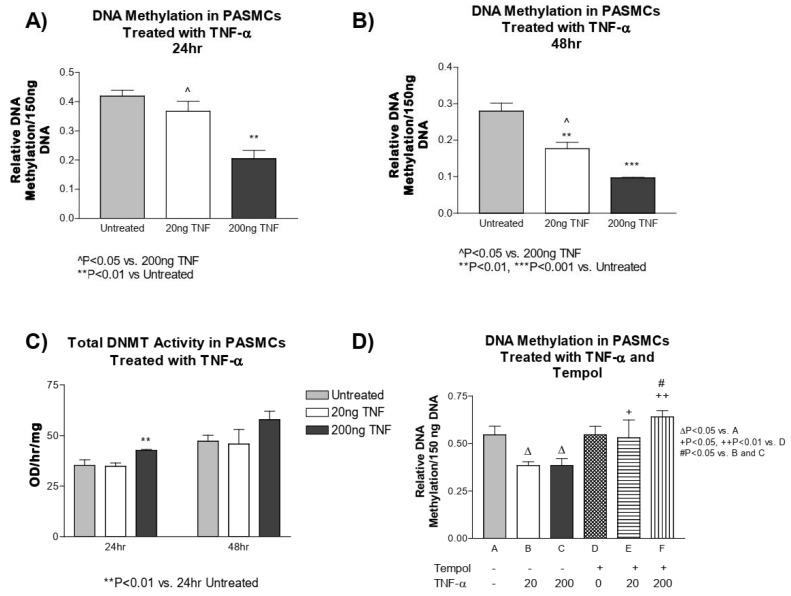
**TNF-α treatment decreases DNA methylation in PASMCs**. DNA was purified from PASMCs treated with or without recombinant TNF-α (rTNF-α) for 24 or 48 h and the global DNA methylation was measured using an ELISA-based microplate assay that bound methylated DNA. (**A**) DNA methylation after 24 h of treatment. (**B**) DNA methylation after 48 h of treatment. (**C**) DNMT1 activity. (**D**) Cells were then pre-treated with tempol (1 mM) for 24 h prior to rTNF-α treatment for 24 h. DNA methylation was measured using the same method as described above. *n* = 3 independent replicates. ** *p* < 0.01 and *** *p* < 0.001 vs. untreated. ^ *p* < 0.05 vs. 200 ng TNF. ^∆^ *p* < 0.05 < 0.01 vs. (**A**); ^+^ *p* < 0.05, ^++^ *p* < 0.01 vs. (**D**); ^#^ *p* < 0.05 vs. (**B**,**C**).

We next investigated whether TNF-α treatment alters the methylation status of DNA in PASMCs. PASMCs treated with TNF-α had a significant decrease in methylated DNA compared to the untreated controls at both 24 and 48 h (Figure 4A,B). We next tested whether the decrease in DNA methylation in PASMCs is a result of decreased DNMT1 enzyme activity. This was not the case, as the DNMT1 activity was slightly increased at 24 h post treatment with a high dose of TNF-α and did not appear to be different at 48 h post treatment with either TNF-α dose (Figure 4C).

Furthermore, to investigate whether superoxide alters DNA methylation, we assessed the DNA methylation level in PASMCs pre-treated with tempol. Again, we found that TNF-α treatment decreased DNA methylation, while tempol treatment, prevented the TNF-α-induced decrease in DNA methylation, and maintained it at a level similar to the untreated controls (Figure 4D). This result suggests that the TNF-α-induced decrease in DNA methylation is mediated by superoxide in PASMCs.

### 3.6. GADD45-α siRNA Does Not Prevent the TNF-α-Induced Decrease in DNA Methylation

**Figure 5:**
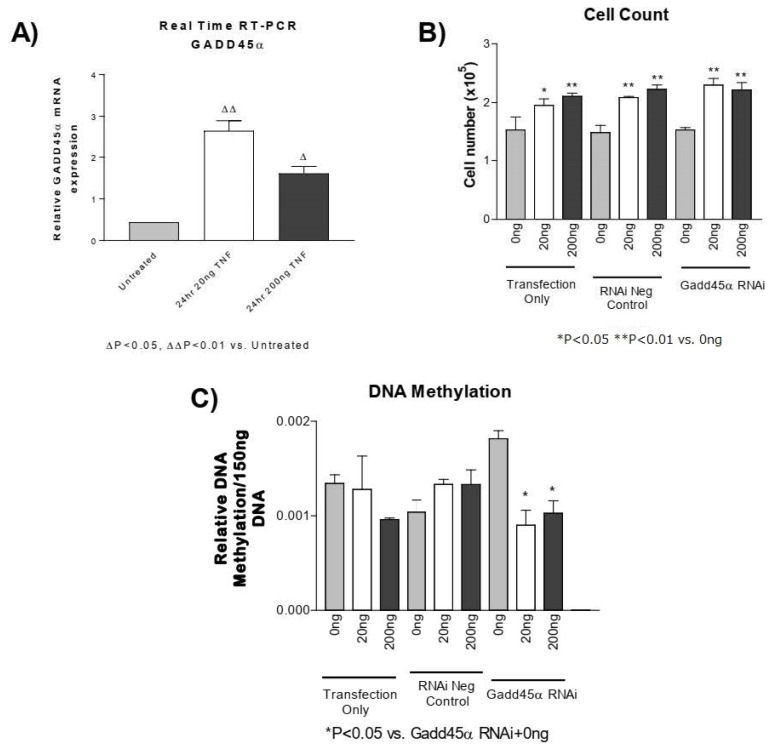
**GADD45-α siRNA does not prevent the TNF-α-induced decrease in DNA methylation.** (**A**) Real-time reverse transcription PCR was used to determine GADD45-α mRNA in PASMCs treated with rTNF-α for 24 or 48 h. PASMCs were treated with GADD45-α siRNA, negative siRNA, or lipofectamine only for 24 h prior to rTNF-α treatment for 24 h. ^∆^ *p* < 0.05, ^∆∆^ *p* < 0.01 vs. untreated; (**B**) Cell proliferation. (**C**) DNA methylation. *n* = 3 independent replicates. * *p* < 0.05 and ** *p* < 0.01 vs. GADD45 RNAi + 0 ng.

GADD45-α is a potential regulator of DNA demethylation [51,52,53]. Using real-time PCR, we found that the GADD45-α mRNA level was significantly elevated following TNF-α treatment in PASMCs (Figure 5A). Knockdown of GADD45-α using siRNA significantly decreased GADD45-α mRNA (Figure S2), which increased DNA methylation (Figure 5C). Knockdown of GADD45-α, however, did not prevent either the TNF-α-induced increase in PASMC proliferation (Figure 5B) or the TNF-α-induced decrease in DNA methylation (Figure 5C). These results suggest that increased GADD45-α may not be involved in TNF-α-induced decrease in DNA methylation or PASMC proliferation.

### 3.7. TNF-α Treatment Decreases H3-K4 Methylation in PASMCs

**Figure 6:**
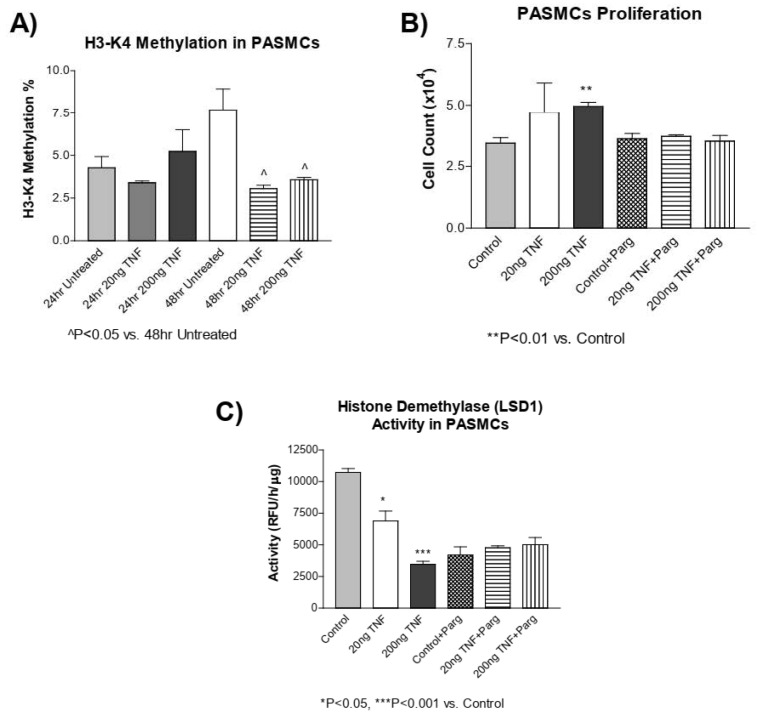
H3-K4 methylation is decreased in rTNF-α treated PASMCs, which is not prevented by pargyline, an LSD1 demethylase inhibitor. (**A**) H3-K4 methylation was measured in PASMCs treated with rTNF-α for 24 or 48 h. (**B**) Cell proliferation was determined in PASMCs co-treated with pargyline, a LSD1 inhibitor, and rTNF-α. (**C**) LSD1 activity was determined in PASMCs co-treated with pargyline and rTNF-α. *n* = 3 independent replicates. * *p* < 0.05, ** *p* < 0.01 and *** *p* < 0.001 vs. Control. ^ *p* < 0.05 vs. 48 h Untreated.

DNA methylation and histone methylation mechanisms have been shown to interact with one another [1]. Thus, we assessed whether TNF-α treatment alters histone methylation in addition to DNA methylation. Interestingly, TNF-α treatment significantly decreased H3-K4 methylation at 48 h (Figure 6A). The histone demethylase enzyme, LSD1, regulates H3-K4 methylation. Using pargyline, a LSD1 inhibitor [54], we tested whether co-treatment with pargyline could affect TNF-α-induced PASMC proliferation. PASMC proliferation was increased by TNF-α treatment and inhibition of LSD1 by pargyline prevented PASMC proliferation due to treatment with TNF-α (Figure 6B) in cells treated with TNF-α. This result suggests that TNF-α-induced PASMC proliferation may involve LSD1. LSD1 activity was decreased by TNF-α treatment, while pargyline inhibited LSD1 activity in cells treated with or without TNF-α (Figure 6C). This result suggests that pargyline and TNF-α may act on the same pathway. An additional study is needed to investigate whether TNF-α affects the H3-K4 methyltransferase activity. Collectively, these results suggest that in addition to DNA methylation, the histone methylation mechanism may be involved in PASMC proliferation.

## 4. Discussion

Our recent study showed that cold exposure initiates an early and robust increase in the pro-inflammatory cytokine TNF-α in pulmonary arteries and lungs [5]. TNF-α contributes to cold-induced PA hypertrophy and pulmonary hypertension (PH) [5]. An increase in TNF-α was also found in other models of PH [1,55,56], as well as in PH patients [26,27]. In this study, we found that TNF-α increased superoxide production in isolated PASMCs, likely via upregulation of NADPH oxidase activity (Figure 1). NADPH oxidase is the major source of superoxide in the vascular cells [1,57]. To our knowledge, this study provides the first evidence that TNF-α decreased DNA methylation and increased cell proliferation (Figure 2, Figure 3, Figure 4 and Figure 5), a hallmark of PA remodeling, in PASMCs. These responses may be partly associated with increased superoxide because quenching of superoxide by tempol prevented TNF-α-induced DNA hypomethylation and cell proliferation in PASMCs (Figure 3 and Figure 4). On the other hand, tempol can increase H_2_O_2_ production [58], which may in turn inhibit cell proliferation. Thus, we cannot exclude the possibility that the effect of tempol on TNFα-induced PAMSC proliferation may also be partially attributed to the H_2_O_2_ generation. It was reported that TNF-α can inhibit PASMC PDH activity and induce a PAH phenotype [56]. It is new and interesting that superoxide downregulates DNA methylation in PASMCs. The finding that a decrease in DNA methylation contributes to cell proliferation provides a new direction for addressing PA remodeling and PH.

We further investigated whether epigenetic mechanisms were altered in TNF-α-induced PASMC proliferation. Unexpectedly, the acetylation status of histone H3 was not altered by TNF-α (Figure S1A–C). We next determined the methylation status of DNA isolated from TNF-α-treated PASMCs. Treatment with TNF-α for both 24 and 48 h reduced the global methylation status of PASMCs and, importantly, this was prevented by pre-treatment with tempol (Figure 4A–D). A decrease in DNA methylation is normally associated with an increase in gene transcription and replication. If the DNA was hypomethylated in the promoter or enhancer-related region of a gene (or genes) involved in proliferation or anti-apoptosis, then sustained gene expression could result in PASMC proliferation and occlusion of the pulmonary vasculature, contributing to the development of PH. Further work is needed to identify specific hypomethylated genes that contribute to the proliferation of PASMCs.

Unlike histone demethylases, which remove methylated lysines on histone tails, there are no known enzymes that are directly responsible for the demethylation of DNA. However, several mechanisms associated with demethylation of DNA involve either base excision or nucleotide excision repair pathways, where a methylated base is removed and replaced with a new and nonmethylated base [59,60,61]. To explore the potential mechanism of TNF-α-induced DNA hypomethylation, we measured the mRNA of several candidate base repair genes associated with the demethylation of DNA (Supplemental Table S1). Among these genes, growth arrest and DNA damage inducible-α (GADD45-α) expression were clearly increased by TNF-α treatment (Figure 5A). GADD45-α belongs to a family of enzymes that are implicated in a variety of cellular processes including cell cycle arrest, DNA repair, and apoptosis in response to physiological and environmental stresses [62]. Furthermore, GADD45-α has been demonstrated to interact with PCNA and promote nucleotide excision repair pathways supporting a role in DNA demethylation [51,52,53]. The established role for GADD45 proteins includes the promotion of DNA repair and tumor suppressor properties but the function of GADD45-α is highly dependent on expression level, cellular localization, and post-translational modifications [62]. Contrasting data suggest that GADD45-α can act as a tumor promoter, as altered expression of GADD45-α protein has also been found in several solid tumors and hematopoietic malignancies involving proliferation [62,63]. In our experiment, the knockdown of GADD45-α using siRNA did not appear to prevent the TNF-α-induced increase in cell proliferation or decrease in DNA methylation. It is possible, then, that the increase in GADD45α in response to TNF-α may not be promoting a decrease in DNA methylation and that it may be a compensatory response intended to promote DNA repair and prevent chromosomal instability. One possible way to address this conclusion that was beyond the scope of this study would be to overexpress GADD45-α in PASMCs treated with TNF-α to determine whether it is in fact a beneficial response.

Lastly, we investigated whether histone methylation may be contributing to the decrease in DNA methylation. Recent evidence suggests that DNA methylation and histone methylation mechanisms may be regulated, or affected, by one another [64]. For example, in *Arabidopsis*, DNMT1 mutations result in altered patterns of other epigenetic marks, including methylation of lysines on histone proteins [65], and in *Neurospora crassa*, the loss of H3-K9 methyltransferase leads to a loss of DNA methylation [66]. Collectively, this and other data support the hypothesis that there is cross-talk between histone methylation and DNA methylation pathways. We found that TNF-α treatment decreased the methylation of H3-K4, which is supported by previous observations that methylation patterns of both DNA and histones contribute to cell proliferation [67,68,69]. We noticed that the H3-K4 methylation levels were not affected by an H3-K4 demethylase (LSD1) inhibitor, pargyline, although it prevented PASMC proliferation (Figure 6). Unexpectedly, the LSD1 activity was decreased by TNF-α treatment, which is the opposite of our hypothesis. While it was reported that LSD1 regulates the expression of pro-inflammatory cytokines [70], this is the first report that the LSD1 activity was decreased in response to TNF-α. Collectively, however, the evidence suggests that the histone methylation mechanism may be involved in PASMC proliferation but further work is needed to investigate the underlying mechanisms.

## 5. Perspective

**Figure 7:**
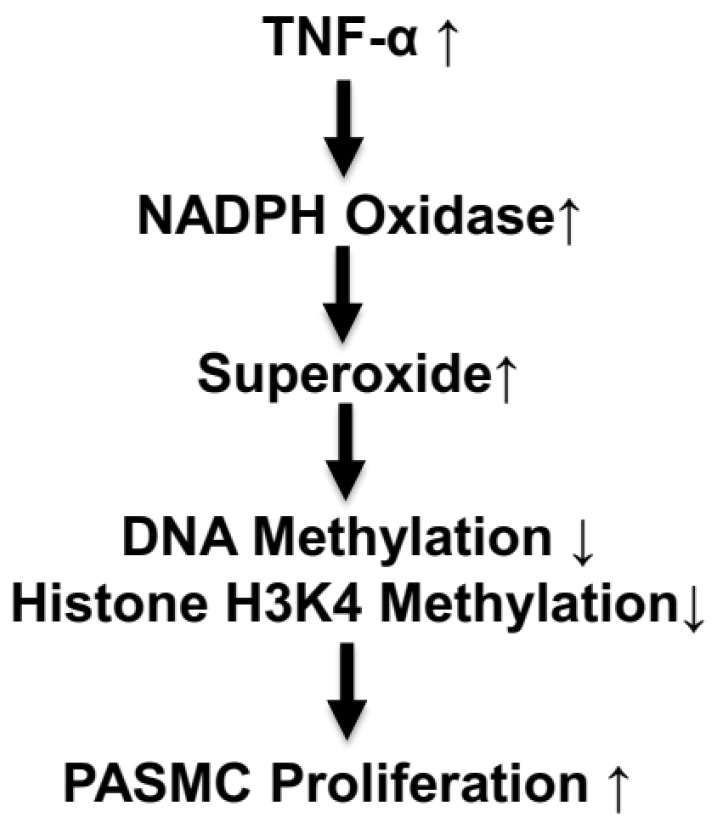
Schematic diagram illustrating the epigenetic pathway in TNF-α-induced PASMC proliferation.

This study showed for the first time that the TNF-α-induced increase in superoxide caused a decrease in DNA methylation and an increase in cell proliferation in isolated PASMCs (Figure 7). These findings are significant because they provide a potential new direction for further investigating PASMC proliferation in PH pathogenesis. Additional work, however, is needed to identify specific hypomethylated genes that regulate PASMC proliferation. This study provided evidence that TNF-α downregulated histone H3K4 methylation, which may be involved in PASMC proliferation. The relationship between DNA/histone methylation and PASMC proliferation could be a valuable novel epigenetic pathway to explore not just PH pathogenesis but also other vascular remodeling diseases.

## Data Availability

The datasets generated during and/or analyzed during the current study are available from the corresponding author upon reasonable request.

## Supplementary Materials

The following supporting information can be downloaded at: https://www.mdpi.com/article/10.3390/antiox13060677/s1. Table S1. Oligonucleotides for real-time reverse transcription–polymerase chain reaction evaluation of DNA demethylase genes. Figure S1. TNF-α treatment does not alter histone acetylation in PASMCs. Histone protein was extracted from PASMCs treated with or without rTNF-α for 24 or 48 h and the global H3 acetylation was determined using an ELISA-based microplate assay that bound acetylated H3 protein. (A) global H3 acetylation at 24 h treatment with rTNF-α, (B) global H3 acetylation at 48 h treatment with rTNF-α, and (C) combined results (24 and 48 h) of global H3 acetylation. Figure S2. GADD45-α siRNA effectively silenced GADD45-α Mrna expression. Real time reverse transcription PCR was used to determine GADD45-α mRNA in PASMCs treated with rTNF-α (200 ng) for 24 h. PASMCs were treated with GADD45-α siRNA, negative siRNA, or lipofectamine only for 24 h prior to rTNF-α treatment for 24 h. *n* = 3 independent experiments. ^Δ^ *p* < 0.05, ^ΔΔ^ *p* < 0.01 vs. Transfection only; ** *p* < 0.01 vs. RNAi Neg + 200 ng TNF.
