# Supplementary material for: TNFα Induces DNA and Histone Hypomethylation and Pulmonary Artery Smooth Muscle Cell Proliferation Partly via Excessive Superoxide Formation
Source: Antioxidants (Basel). 2024 May 31;13(6):677. doi: 10.3390/antiox13060677 (PMC11200563; doi:10.3390/antiox13060677)
Supplement: Supplementary file 1 [file antioxidants-13-00677-s001.zip › antioxidants-2985206-supplementary.pdf]

Online Data Supplement

TNFα Induces DNA and histone hypomethylation and Pulmonary Artery Smooth Muscle Cell Proliferation *via* Excessive Superoxide Formation

Patrick Crosswhite[2,3] and Zhongjie Sun[1,2]

[1]Department of Physiology, College of Medicine, University of Tennessee Health Sciences Center, Memphis, TN 38163, USA

[2]Department of Physiology, College of Medicine, University of Oklahoma Health Sciences Center, Oklahoma City, OK 73104, USA

[3]Department of Human Physiology, Gonzaga University, Spokane, WA 99205, USA

Running Title: PASMC Proliferation by TNFα

Total characters: 36,539; **Total Words**: 5,143

Address Correspondence to:
Zhongjie Sun, MD, PhD, FAHA
Professor and Chair
Department of Physiology
College of Medicine
University of Tennessee Health Sciences Center
956 Court Ave, Coleman A302
Memphis, TN 38028
USA
ZSun10@uthsc.edu
Tel. 901-448-2679

Online Supplemental Data

Supplemental Table S1. Oligonucleotides for real-time reverse transcription–polymerase chain reaction evaluation of DNA demethylase genes.

Messenger RNA	Description	Sense	Antisense
Apobec1	Cytidine deaminase enzyme, C-U editing	GCAAGATGAGTTCCGAGACG	AGTTCCCGGGGGTCAAAG
Apobec2	Cytidine deaminase enzyme, C-U editing	CTCCATGGCTCAGAAGGAAG	TCGATCAGCTCCTTCAGCTT
Gadd45α	Growth arrest and DNA damage inducible protein, stress response	AGCCAAGCTGCTCAACGTA	TGAGGGTGAAATGGATCTGC
Gadd45β	Growth arrest and DNA damage inducible protein, stress response	GAATGTGGACCCCGACAG	ACGATTGGATCAGGGTGAAG
MBD2	Methyl CpG binding protein	CTGCATCCATTTTCAAGCAA	CCAGAAAAGCTGACGTGGTT
Tet1	DNA hydroxylase enzyme	AAACGTACCTGCACCTGTCA	TTTTCTGGGGTTTGCACTTC
Tet3	DNA hydroxylase enzyme	CCGAGGTGGAAATAAATGCT	GGAACAGGTCCATCAACTGG
TDG	Thymine-DNA glycosylase	CCGGATCCTGTGCTACTCTC	GAGCTTCTGCAGGCATCTG

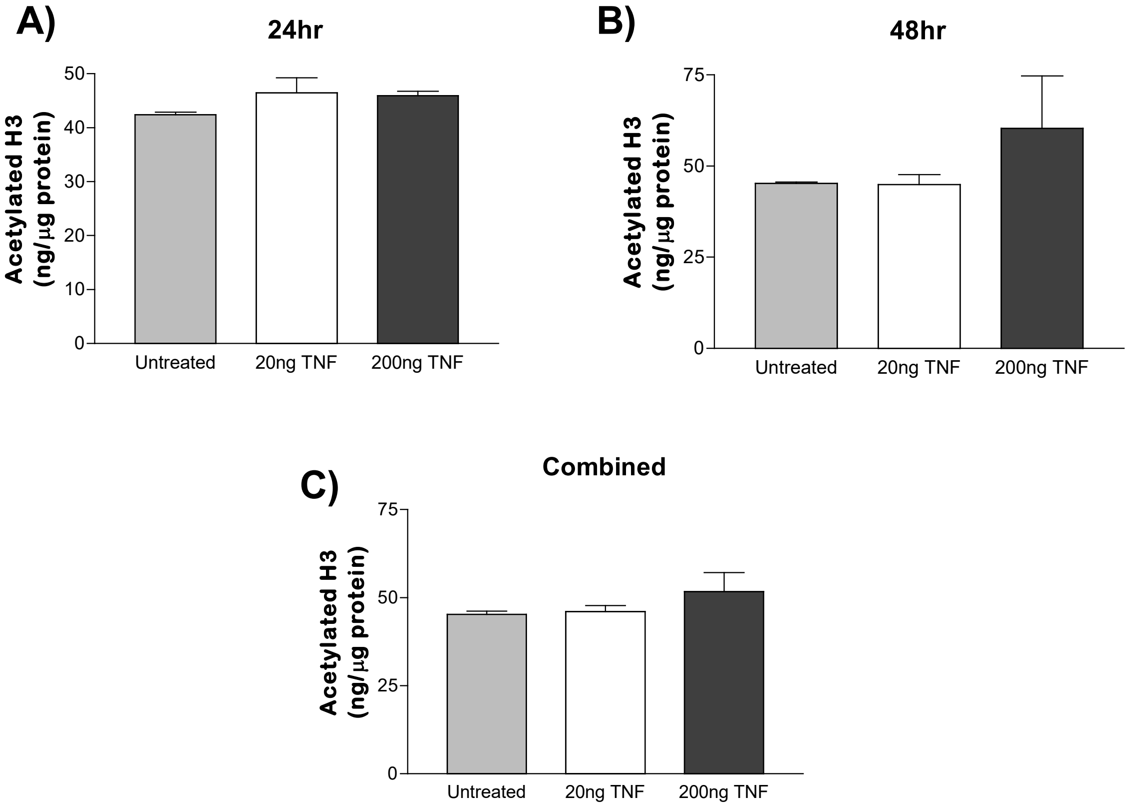

Supplemental Figure S1. TNF-α treatment does not alter histone acetylation in PASMCs. Histone protein was extracted from PASMCs treated with or without rTNF-α for 24 or 48 hours and the global H3 acetylation was determined using an ELISA-based microplate assay that bound acetylated H3 protein. **A)** global H3 acetylation at 24 hrs treatment with rTNF-α, **B)** global H3 acetylation at 48hrs treatment with rTNF-α, and **C)** combined results (24 and 48hrs) of global H3 acetylation.

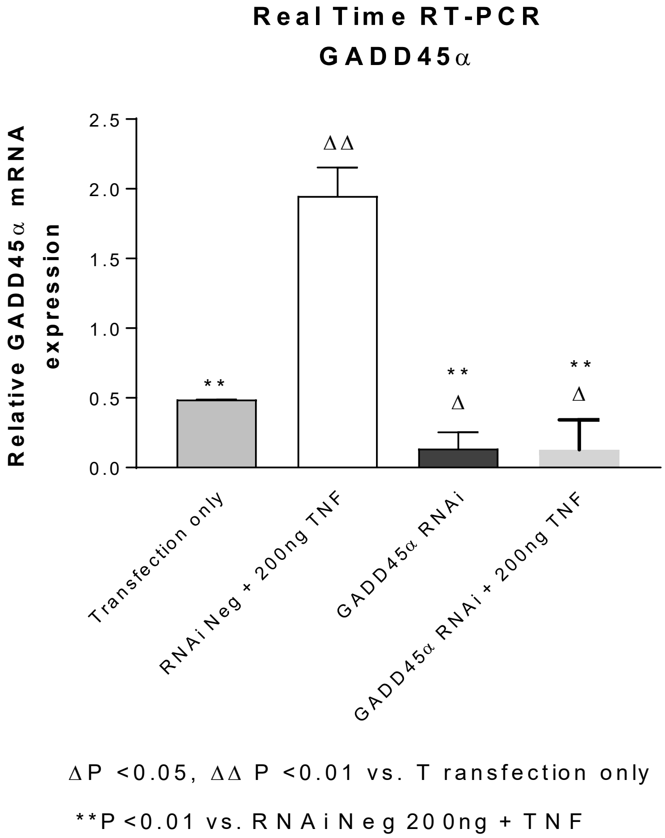

Supplemental Figure S2. GADD45-α siRNA effectively silenced GADD45-α mRNA expression. Real time reverse transcription PCR was used to determine GADD45-α mRNA in PASMCs treated with rTNF-α (200 ng) for 24 hours. PASMCs were treated with GADD45-α siRNA, negative siRNA, or lipofectamine only for 24 hours prior to rTNF-α treatment for 24 hours. N=3 independent experiments. $^{\Delta}P<0.05$, $^{\Delta\Delta}P<0.01$ *vs.* Transfection only; $^{**}P<0.01$ vs. RNAi Neg + 200ng TNF.
